# Assessment of Quality of Life of Epileptic Patients in Ethiopia

**DOI:** 10.1155/2020/8714768

**Published:** 2020-01-02

**Authors:** Esileman Abdela Muche, Mohammed Biset Ayalew, Ousman Abubeker Abdela

**Affiliations:** Department of Clinical Pharmacy, School of Pharmacy, College of Medicine and Health Sciences, University of Gondar, Gondar, Ethiopia

## Abstract

**Background:**

Patients with epilepsy are at an increased risk of poor quality of life.

**Purpose:**

We aimed at assessing the quality of life and its determinants among epileptic patients at University of Gondar Referral Hospital (UoGRH), Ethiopia.

**Methods:**

Institution based cross-sectional study was conducted on epileptic patients on follow up at UoGRH from January 15 to April 15, 2017. Information including socio-demographic profile and diagnosis was extracted from medical records and patients. Quality Of Life In Epilepsy-10 (QOLIE-10) tool was used to measure the quality of life. Independent *t*-test and one-way analysis of variance were used to look for factors associated with quality of life. The level of statistical significance was declared at *P*-value ≤ 0.05.

**Results:**

A total of 354 patients were included in the study and mean age was 29.1 ± 11.7 years. The mean QOLIE-10 score was 19.85. One hundred ninety-four (54.8%) of participants had a good quality of life. Being illiterate, unemployment, and presence of co-morbid medical condition were associated with poorer quality of life.

**Conclusion:**

Nearly half of the participants had a poor quality of life. Patients with co-morbidity, illiteracy, and unemployment should be given special emphasis in order to improve their quality of life.

## 1. Introduction

The International League Against Epilepsy (ILAE) defines epilepsy as a disease of the brain defined by any of the following conditions: (i) At least two unprovoked (or reflex) seizures occurring > 24 h apart; (ii) one unprovoked (or reflex) seizure and a probability of further seizures similar to the general recurrence risk (at least 60%) after two unprovoked seizures, occurring over the next 10 years; or (iii) diagnosis of an epilepsy syndrome [1].

Epilepsy affects people of any age, gender, ethnicity, and social background, irrespective of geographic location. It is the most common chronic serious neurologic disease [2]. About 10% of the whole world population living a normal life span can expect to have at least one epileptic seizure. At least 50 million will have recurrent seizures. This could be underestimated, as partial seizures are often not recognized as such in the developing world [3]. The point prevalence of active epilepsy was 6.38 per 1,000 persons while the lifetime prevalence was 7.60 per 1,000 persons [4]. In a large community-based epidemiological study, the prevalence of epilepsy in Ethiopia was reported as 5.2 per 1000 population. The incidence was 64 per 100,000 population [5].

Growing recognition of the importance of the psychosocial effects of epilepsy has led to the need to quantify the quality of life in affected individuals. Patients with epilepsy are at increased risk of poor Quality of Life (QOL) [6, 7]. WHO defines quality of life as the perception that an individual has of his place in existence, in the context of culture and system securities in which they live, and in relation to their goals, expectations, standards, and concerns [8]. Different tools were used to standardize this concept and many studies evaluated the quality of life of epileptic patients using various types of questionnaires. QOLIE-10 questionnaire, which was used in this study, is a valid and reliable tool used to measure the QoL of patients with epilepsy which was validated by Cramer et al. [9].

People with epilepsy face a condition that can affect their QoL in multiple domains. These include physical (increased risk of injury and death), psychological (increased risk of anxiety and depression), cognitive (both epilepsy and the medications used to treat it are associated with impaired cognition), and social and occupational (epilepsy is a stigmatizing condition, and also often carries limitations on driving and employment) [10]. Hence, assessment of QoL is important in the management of epilepsy to achieve the optimal goal of therapy.

The existing studies also revealed that individuals with epilepsy suffer from a number of social, psychological and physical poor outcomes. For example, a study conducted in Ethiopia revealed that 60% of the study participants faced different problems due to their illness such as stigma (24%), inability to find a partner (31%), educational problems (17%), and problems of employment (9%)[11]. The recently released Ethiopian mental health strategic plan emphasizes the special considerations to be given to these vulnerable patient groups [12].

Though different studies have been conducted in different parts of the world including Africa, very few studies were undertaken on quality of life of epilepsy patients in Ethiopia. The evidence for the different socio-demographic characteristics like level of education, age, marital status, employment status and some clinical characteristics of epileptic patients as a factor for QoL is inconsistent [13]. The aim of this study was to determine the quality of life of epileptic patients who were taking anti-epileptic drugs and to identify factors associated with it. This study can help as an input for hospital managers' performance measurement and to improve treatment outcomes of epileptic patients. It can also be used as baseline information for researchers who need to conduct further study in the area.

## 2. Materials and Methods

### 2.1. Study Area and Period

The study was conducted at University of Gondar Referral Hospital, North-West Ethiopia. It is one of the biggest tertiary level referral and teaching hospital in the region. It provides service for an estimated 7 million population. The hospital serves as a referral center for North Gondar administrative region and the residents around. It has 400 beds in five different inpatient departments and 14 wards. Patients with Neurologic disorders get service two days per week. The service is given by two neurologists. There are around 2200 epileptic patients on follow up. Data was collected from January 15 to April 15, 2017.

### 2.2. Study Design and Subjects

We employed hospital-based prospective cross sectional study. Adult epileptic patients who were on follow up in UoGRH were included as a study subject. We excluded epileptic patients who were less than 18 years old, those who were on antiepileptic drugs (AEDs) for less than one year period, patients with incomplete information on their chart and involuntary and uncooperative patients.

### 2.3. Sample Size & Sampling Methods

The sample size was calculated using single population proportion formula, where *Z* = 1.96, *α*/2, *P* = 54.2%, taken from a study done on similar setting [14], and 5% for *d* (margin of error). The total sample size calculated was 381. Since the source population were <10,000 the sample was recalculated using correction formula which gave the sample size of 324. After adding 10% contingency the final sample size became 356. We used systematic random sampling to recruit participants in each data collection day. The sampling fraction (*k*) was calculated through dividing the number of study population available each day by the maximum possible number of patients' that could be interviewed the same day. Every *k*^th^ patient was then interviewed following physician's visit.

### 2.4. Data Collection Instrument

Data was taken from patients' medical chart and patient interview using data extraction format; which was pretested in 18 patients (5% of calculated sample size) in Felegehiwot referral hospital. Little adjustment was made to clarify questions. QOLIE-10 questionnaire was used to assess quality of life of epileptic patients [9]. There were 10 questions about health and daily activities, one question about how much distress they feel about problems and worries related to epilepsy, and a review of what bothers them most. Two individuals who were fluent in both the English and Amharic languages translated the QOLIE-10 into Amharic version. The new Amharic version of QOLIE-10 was back-translated into English to ensure that the meaning and comprehension of the original version was retained. The Amharic version of the questionnaire was also checked for both the accuracy and meaning of the translated versions before finalized.

### 2.5. Data Collection Procedures

Six BSc nurses who had basic knowledge of epilepsy treatment collected the data from January 15 to April 15, 2017. Patient demographics, diagnosis, co-morbid conditions and type of drug were collected on the patient charts by using well designed data extraction format. Information needed to measure patients' quality of life was collected through face to face interview using Amharic version of QOLIE-10 questionnaire. Data collectors were trained for 2 days on the documentation and techniques of data collection. Medical chart number was documented to avoid repetition of participants. The principal investigators reviewed all filled data abstraction formats daily.

### 2.6. Data Processing and Analysis

Data was entered into SPSS version 20.0 for analysis. We applied double entry to check whether the data was entered correctly or not. Descriptive statistics was used for demographic details and inferential statistical tests like the *t*-test for independent variables. Analysis of variance (ANOVA) was used to compare QOL scores among various socio-demographic factors and clinical characteristics. Level of statistical significance was declared at *P*-value ≤ 0.05. Quality of life was considered as poor If QOLIE-10 Score for individual patient was less than the mean QOLIE-10 score for the study population.

The overall quality of life score represent the summation of all 10 item questions in the QOLIE-10 instrument. The questionnaire used has minimum score of 10 and maximum score of 50. Higher score indicate poor QoL and the lower the score the better QoL.

### 2.7. Ethical Consideration

We obtained letter of ethical clearance from ethical review board of school of pharmacy and letter of cooperation from UoGRH hospital. Each participant was informed about the objective of the study, procedures of selection and assurance of confidentiality. Oral consent was taken from participants before data collection. Privacy and confidentiality were ensured during patient interview and review of patients chart. Name and address of the participants were not recorded in the data extraction formats.

## 3. Results

### 3.1. Socio-Demographic Characteristics of Study Subjects

Out of the total 356 epileptic patients enrolled, 354 of them completed the interview and included in the analysis. The mean age of participants was 29.1 ± 11.7 years with a range of 18–88 years. More than half (61%) of respondents were male, quarter of the participants (25%) were married, 209(59%) were from urban area, 218(61.6%) of respondents were below primary school in educational background and most of the participants (89.7%) were orthodox Christian in religion. One third of participants were students, 70.6% had below 66 USD household monthly incomes. The detailed description of socio-demographic characteristics of the study participants is shown in Table 1.

### 3.2. Clinical Characteristics of the Study Subjects

Most of the patients (63.8%) had at least one episode of seizure a year before. The mean age of onset of epilepsy was 22 ± 12.1 years, the mean duration of epilepsy was 7.8 ± 6.4 years. Most (82.2%) of participants were on a single AED (Table 2).

### 3.3. Participants' Response to Quality of Life Assessment Questions

Participants mean quality of life in epilepsy-10 (QOLIE-10) score was 19.86 ± 6.91, with a minimum and maximum score of 10 and 40 respectively. Equivalent mean quality of life result was 75.36%. One hundred ninety four (54.8%) of participants had a good quality of life. Two hundred sixty one (73.7%) of participants had enough energy for most of their time, but 180(50.85%) of the patients feel down hearted or blue for at least some of their time. For more than half (54%) of patients, their epilepsy and or AEDs didnot terribly disturb their daily life (Table 3).

### 3.4. Factors Associated with Quality of Life

One way ANOVA analysis of QOLIE-10 scales with socio demographics and clinical characteristics showed that educational status, occupation, income, frequency of seizure during last follow up, frequency of seizure during a year before, number of AEDs and patient's perception of current status of epilepsy have significant association with quality of life.Independent *t*-test also showed that presence of co-morbidity and methods of acquiring AEDs, were significantly associated with quality of life (Table 4).

*Note:* independent *t*-test was done for variables with 2 categories and one way ANOVA was done for those variables with 3 or more categories.

After detecting the presence of association between some of the patients' characteristics and quality of life using one way ANOVA, then Post hock analysis was done to determine where the significant mean difference lies. Accordingly illiterates, unemployed individuals and patients with less than 22 USD monthly incomes had significantly poorer quality of life as compared to their counter parts. Patients who perceive their epilepsy status was controlled had better quality of life as compared to patients who thought that it was improved, deteriorated or had no change (Table 5).

## 4. Discussion

The mean QOLIE-10 score in this study was 19.86 (75.3%). This result is in line with the study in Bangalore, India, which reported a mean quality of life of 74.9% [15]. However this finding was higher than many other studies conducted in different part of the world [14,  16–20]. This difference may be explained by the presence of high percentage of two or more AEDs use in those studies in contrast to the finding in the current study. Different literatures suggested that patients receiving two or more AEDs had poor quality of life as compared to patients on monotherapy [14, 21–24].

The higher rate of monotherapy (82.2%) reported in this study is in line with the result of previous studies done in Ethiopia. The study conducted on two hospitals in Northwest Ethiopia reported that 76.7% of epileptic patients were on monotherapy [25]. Another study in Bishoftu general hospital also reported monotherapy users to be 78.6% [26] and a study done by Birru et al. also found that 80.35% [27] of studied patients used single drug for treatment of their epilepsy. However Gurshaw et al. reported only 54.5% of studied epileptic patients in Jimma university hospital used monotherapy [28].

In this study, various socio-demographic and clinical characteristics were checked for their possible influence on quality of life. Significantly poor QoL was seen among people who had no formal education. This finding is supported by many other studies [16, 29–31]. The poor QoL observed in illiterate patients may be due to the lesser knowledge they may have about the diseases and its treatment; as well as they may not easily understand instructions given from health professionals and this may result in poor adherence to medication and poorer seizure control which may finally lead to poor quality of life.

Marital status did not significantly influence QoL. Although this result is in agreement with the study done in India [15], it is contrasting with the result of the studies conducted in Malaysia, Iran and China. [31–33]. Quality of life of unemployed patients is significantly lower than those of epileptic patients who have a job. This may be because job gives mental satisfaction. Those who are unemployed get bored and face more cognitive problems. This finding is similar to the study done by Singh and Pandey [31] and Ashjazadeh et al. [32]. Mean QOLIE-10 score of patients with household monthly income <22 USD was 21.4. The score is significantly higher (lower quality of life) than for patients who got 22 USD or more per month. Therefore, income significantly affected quality of life of epileptic patients. This is consistent with the result of the study done from the US Centers for Disease Control and Prevention Managing Epilepsy Well Network [34].

Patients who have access to AEDs free of charge and those who purchase their drug out of pocket had mean QOLIE-10 score of 20.97 and 19.12 respectively. Independent *t*-test result showed there is significant difference in QOL score between these 2 groups. Accordingly to those who spent money to buy drug have better quality of life than who get freely. The possible explanation for this may be those who spent money to buy drugs may have better medication adherence than patients who got drugs freely.

Frequent seizure during last follow up and a year before leads to poorer quality of life score as compared to no seizure. Literatures support the finding of this study in which people with frequent seizures had significantly poorer health related QOL than those with infrequent or no seizures [15, 16, 34–36]. This finding emphasizes the importance of encouraging patients to observe symptoms of the onset of a seizure (aura, tingling, numbness, headaches, confusion, etc.), record them, and share the information with their health-care provider. Health education interventions involving medication intake, diet, regular sleep, exercise, and stress reduction can all aid in reducing seizure frequency so that patients will have better quality of life.

Patients who were taking two or more AEDs had lower quality of life as compared to patients who took single AED. Similar result was found in many other previous studies [14, 15, 21–24, 37]. Patients who perceive that their current status of epilepsy was controlled had better quality of life than patients who perceive that their epilepsy was improving, had no change or had deteriorated. In addition patients who perceived their disease is improving had better quality of life than those who perceived nothing is changed. As patients perceive their epilepsy is controlled or improving, their worry about the illness and the fear of seizure recurrence will reduce and patient will have improved quality of life. Patients who had co morbid diseases had a worse quality of life than those who have only epilepsy. Similar association was reported by Tegegne et al. [26].

Age, gender, place of residency, religion, age at diagnosis of epilepsy and duration on AEDs had no significant association with quality of life. Similar nonsignificant association of Patient's age, age at onset of epilepsy and duration of AEDs with quality of life was reported by Tegegne et al. [26]. Patients' gender did not significantly affect quality of life (*p* = 0.371). Similar result was reported by Singh and Pandey [31].

Even though the use of validated tool, excellent response rate and sufficient sample size were the strengths of this study, it is not without limitation. The cross-sectional nature of this study did not allow establishing casual relation between quality of life and the different factors associated with it. The participants were recruited from one medical center in Northwest Ethiopia. Therefore, the findings may not be generalizable to all PWE in Ethiopia. Although the concept of QOL is very broad and can be influenced by multiple variables, some other clinical and socio-demographic conditions were not addressed in the study (e.g., severity of seizures, anxiety disorders, sleep disorder, specific structural/metabolic cause of epilepsy, and family support, among others) which could affect QOL.

## 5. Conclusion

Nearly half of participants had poor quality of life. Presence of co-morbidity, usage of two or more AEDs, illiteracy, unemployment and monthly income of less than 22 USD were significantly associated with poor quality of life. Patients with such characteristics should be given special emphasis to improve their quality of life. Patients perception of their epilepsy status as controlled or improved, absence of seizure episode during the past 1 year period and lesser number of seizure episodes during the last follow up were significantly related with a better quality of life.

## Figures and Tables

**Table 1:** Socio-demographic characteristics of study participants, UoGRH, 2017.

Variables	Categories	Frequency(*n*)	Percent (%)
Age	18–25	167	47.2
	26–44	150	42.4
	>45	37	10.5

Sex	Male	216	61.0
	Female	138	39.0

Marital status	Single	224	63.3
	Married	89	25.1
	Divorced	31	8.8
	Widowed	10	2.8

Religion	Orthodox	317	89.7
	Protestant	7	1.9
	Muslim	28	7.9
	Jehovah	2	.5

Educational status	Illiterate	107	30.2
	Primary school	111	31.4
	Secondary school	84	23.7
	Diploma and above	52	14.7

Residency	Rural	145	41.0
	Urban	209	59.0

House hold income	<22$^∗^	114	32.2
	22–66$	136	38.4
	66.1–132$	72	20.3
	132.1–220$	24	6.8
	≥220.1$	8	2.3

Occupation	Unemployed	54	15.3
	House wife	28	7.9
	Merchant or private worker	53	15.0
	Student	96	27.1
	Daily laborer	25	7.1
	Farmer	56	15.8
	Others	42	11.9
	Purchase	212	59.9

^∗^$ = USD (United States Dollar).

**Table 2:** Clinical characteristics of study participants at UoGRH; Northwest Ethiopia, 2017.

Clinical character	Category	Frequency (*n*)	Percent (%)
Age at diagnosis	≤15 years old	104	29.4
	16–30 years old	194	54.8
	31–45 years old	34	9.6
	>46 years old	22	6.2

Types of seizure/epilepsy	Focal seizure	5	1.4
	Generalized tonic-clonic epilepsy	305	86.2
	Unclassified epileptic	44	12.4

Number of AEDs	Monotherapy	291	82.2
	Two drug combination	62	17.5
	Three drug combination	1	0.3

Presence of comorbidity	Yes	36	10.2
	No	318	89.8

Duration of epilepsy	<3 years	105	29.7
	3–5 years	69	19.5
	6–10 years	89	25.1
	>10 years	91	25.7

Frequency of seizure during last follow up	No seizure	250	70.6
	1 or 2 seizure	79	22.3
	3–5 seizure	15	4.2
	>5 seizure	10	2.8

Frequency of seizure during last year	Free of seizure	129	36.4
	1–5 seizure	193	54.5
	6–10 seizure	20	5.6
	>10	12	3.4

Current status of epilepsy	Controlled	129	36.4
	Improving	199	56.2
	No change	20	5.6
	Deteriorated	6	1.7

**Table 3:** Response of patients for quality of life assessment questions, UoGRH, Northwest Ethiopia; 2017.

QOLIE-10 questions	All the time *N* (%)	Most of the time *N* (%)	Sometimes *N* (%)	Rarely *N* (%)	Never *N* (%)
Did you have enough energy for the last month?	151 (42.6)	101 (28.5)	47 (13.3)	25 (7)	30 (11.9)
Have you felt down-hearted and blue?	28 (7.9)	64 (18)	88 (24.9)	54 (15.3)	120 (33.9)

	Not at all *N* (%)	A little *N* (%)	Somewhat *N* (%)	A lot *N* (%)	A great deal *N* (%)
Has your epilepsy/AED caused your daily life to be terrible?	191 (54)	61 (17.2)	50 (14.1)	37 (10.5)	15 (4.2)
How much are you bothered by memory difficulty	193 (54.5)	44 (12.4)	60 (17)	40 (11.3)	17 (4.8)
How much are you bothered by work limitation?	189 (53.4)	49 (13.8)	61 (17.2)	43 (12.2)	12 (3.4)
How much are you bothered by social limitation?	214 (60.5)	41 (11.6)	39 (11)	30 (8.5)	28 (7.9)
How much are you bothered by physical effect of AED?	280 (79.1)	18 (5.1)	22 (6.2)	14 (3.9)	20 (5.6)
How much are you bothered by mental effect of AED?	236 (66.6)	40 (11.3)	34 (9.6)	24 (6.8)	20 (5.7)
How fearful are you of having seizure during the next month?	185 (52.2)	77 (21.7)	31 (8.7)	39 (11)	21 (5.9)

	Very well *N* (%)	Pretty good *N* (%)	Good = bad *N* (%)	Pretty bad *N* (%)	Very bad *N* (%)
How has the quality of your life been during the past 4 weeks? That is, how have things been going for you?	58 (16.4)	170 (48.0)	108 (30.5)	16 (4.5)	2 (0.6)

**Table 4:** Association of socio demographics and clinical characteristics with quality of life of epileptic patients, Northwest Ethiopia; 2017.

Characteristics	Category	Frequency (%)	QOLIE-10 (mean)	*P*-Value
Gender	Male	216 (61)	19.5926	0.371
	Female	138 (39)	20.2681	

Age		29.0 ± 11.7 (mean ± SD)		0.515

Address	Urban	209 (59)	19.5694	0.350
	Rural	145 (41)	20.2690	

Marital status	Single	224 (63.3)	19.7188	0.342
	Married	89 (25.1)	19.3933	
	Divorced	31 (8.8)	21.3226	
	Widowed	10 (2.8)	22.5000	

Educational status	Illiterate	107 (30.2)	21.6636	0.003^∗^
	Primary school	111 (31.4)	19.7387	
	Secondary school	84 (23.7)	19.0357	
	Diploma and above	52 (14.7)	17.7115	

Religion	Orthodox	317 (89.5)	20.0365	0.793
	Protestant	7 (2)	19.7500	
	Islam	28 (7.9)	20.0000	
	Jehovah	2 (0.6)	13.0000	

Patient occupation	Unemployed	54 (15.3)	23.2778	0.003^∗^
	House wife	28 (7.9)	20.6429	
	Merchant or private worker	53 (15.0)	18.9057	
	Student	96 (27.1)	19.3542	
	Daily laborer	25 (7.1)	19.0400	
	Farmer	56 (15.8)	19.8214	
	Others^a^	42 (11.9)	17.8095	

Monthly household income	<22 $^∗^	114 (32.2)	21.4123	0.024^∗^
	22–66$	136 (38.4)	19.4265	
	66.1–132$	72 (20.3)	19.3472	
	132.1–220$	24 (6.8)	17.1250	
	≥220.1$	8 (2.3)	17.7500	

Method of acquiring AED	Free	142 (40.1)	20.9718	0.013^∗^
	Purchase	212 (59.9)	19.1085	

Age of onset	<15 years	104 (29.4)	19.4712	0.859
	16–30	194 (54.8)	19.8918	
	31–45	34 (9.6)	20.5000	
	>46	22 (6.2)	20.3636	

Types of seizure	Focal seizure	5 (1.4)	22.8000	0.469
	GTCS	305 (86.2)	19.7049	
	Unclassified epileptic	44 (12.4)	20.5682	
Duration of epilepsy (years)	<3	105 (29.7)	19.1810	0.554
	3–5	69 (19.5)	19.5652	
	5–10	89(25.1)	20.4157	
	>10	91(25.7)	20.3077	

Number of AEDs for treatment	Mono-therapy	291 (82.2)	19.4330	0.020^∗^
	Two or more drug combination	63 (17.8)	21.8095	
Frequency of seizure during last follow up	No seizure	250 (70.6)	18.6240	0.001^∗^
	1-2 seizure	79 (22.3)	21.1646	
	3–5 seizure	15 (4.2)	28.1333	
	>5 seizure	10 (2.8)	27.9000	

Frequency of seizure during last year	No seizure	129 (36.4)	17.4186	0.001^∗^
	1–5	193 (54.5)	20.7098	
	6–10	20 (5.6)	24.8000	
	>10	12 (3.4)	24.0833	

Presence of co-morbidity	Yes	36 (10.2)	22.5278	0.014^∗^
	No	318 (89.8)	19.5535	

Patient perception of epilepsy status	Controlled	129 (36.4)	17.1938	0.001^∗^
	Improving	199 (56.2)	20.9598	
	No change	20 (5.6)	24.7000	
	Deteriorated	6 (1.7)	24.3333	

^∗^Significant association. GTCS: Generalized tonic clonic epilepsy. $ = USD (United States Dollar).

**Table 5:** Post-hoc analysis of demographic and clinical characteristics of patients which have significant association with quality of life in one way ANOVA, UoGRH, Northwest Ethiopia.

Characteristics	Reference category	Compared with	Mean difference	Standard error	*P* value	95% confidence interval	
Educational status	Illiterate	Primary school	1.92481	.92214	.038	.111	3.738
		Secondary school	2.62784	.99222	.008	.676	4.579
		Diploma and above	3.95201	1.15061	.001	1.689	6.215

Occupation	Unemployed	Merchant or private worker	4.37212	1.31080	.001	1.794	6.950
		Student	3.92361	1.15317	.001	1.655	6.191
		Daily laborer	4.23778	1.63994	.010	1.012	7.463
		Farmer	3.45635	1.29296	.008	.9133	5.999
		Others	5.46825	1.39474	.000	2.725	8.211

Income	<22$^∗^	22–66 $	1.98581	.86855	.023	.2776	3.694
		66.1–132$	2.06506	1.02963	.046	.0400	4.090
		132.1–220$	4.28728	1.53612	.006	1.266	7.308

Patient perception of epilepsy status	Controlled	Improving	−3.76600	.74283	.000	−5.227	−2.305
		No change	−7.50620	1.57928	.000	−10.612	−4.400
		Deteriorated	−7.13953	2.74455	.010	−12.537	−1.741
	Improving	Controlled	3.76600	.74283	.000	2.305	5.227
		No change	−3.74020	1.54154	.016	−6.772	−.708

Frequency of seizure during last year	No seizure	1–5 seizure/year	−3.29124	.75071	.000	−4.767	−1.814
		5–10 seizure/year	−7.38140	1.58636	.000	−10.501	−4.261
		>10 seizure/year	−6.66473	1.99225	.001	−10.583	−2.746

Frequency of seizure during last follow up	1–5 seizure	5–10 seizure/follow up	−4.09016	1.55065	.009	−7.139	−1.040
	No seizure	1 or 2 seizure/ follow up	−2.54056	.83566	.003	−4.184	−.897
		3 up to 5 seizure/follow up	−9.50933	1.72116	.000	−12.894	−6.124
		More than 5 seizure/follow up	−9.27600	2.08800	.000	−13.382	−5.169
	1 or 2 seizures	3–5 seizure/follow up	−6.96878	1.82355	.000	−10.555	−3.382
		>5 seizure/follow up	−6.73544	2.17317	.002	−11.009	−2.461

$ = USD (United States Dollar).

## Data Availability

Data underlying the findings of the study could be found up on reasonable request from the corresponding author.

## Abbreviations

AED: Anti-epileptic drug
ANOVA: Analysis of variance
GTCS: Generalized tonic clonic seizure
ILAE: The international league against epilepsy
OPD: Outpatient department
PWE: Patients with epilepsy
QoL: Quality of Life
QOLIE-10: Quality of life in epilepsy ten
SPSS: Statistical package for social science
UoGRH: University of gondar referral hospital
WHO: World health organization
USD: United States dollar.

## References

[1] Fisher R. S., Acevedo C., Arzimanoglou A. (2014). Practical clinical definition of epilepsy. *Epilepsia*.

[2] Perucca E., Covanis A., Dua T. (2014). Commentary: epilepsy is a global problem. *Epilepsia*.

[3] WHO (2004). *Epilepsy in the WHO African Region: Bridging the Gap. The Global Campaign against Epilepsy*.

[4] Fiest K. M., Sauro K. M., Wiebe S., Patten S. B., Kwon C.-S., Dykeman J. (2017). Prevalence and incidence of epilepsy a systematic review and meta-analysis of international studies. *Neurology*.

[5] Tekle-Haimanot R., Forsgren L., Ekstedt J. (1997). Incidence of epilepsy in rural central Ethiopia. *Epilepsia*.

[6] Gholami A., Salarilak S., Lotfabadi P., Kiani F., Rajabi A., Mansori K. (2016). Quality of life in epileptic patients compared with healthy people. *Medical Journal of the Islamic Republic of Iran*.

[7] Kerr M. P. (2012). The impact of epilepsy on patients’ lives. *Acta Neurologica Scandinavica Supplementum*.

[8] Jacoby A., Snape D., Baker G. A. (2009). Determinants of quality of life in people with epilepsy. *Neurologic Clinics*.

[9] Cramer J. A., Perrine K., Devinsky O., Meador K. (1996). A brief questionnaire to screen for quality of life in epilepsy. The QOLIE-10. *Epilepsia*.

[10] Blond B. N., Detyniecki K., Hirsch L. J. (2016). Assessment of treatment side effects and quality of life in people with epilepsy. *Neurologic Clinics*.

[11] Birhanu S., Alemu S., Asmera J., Prevett M. (2002). Primary care treatment of epilepsy. *Ethiopian Journal of Health Development*.

[12] Fdroemo S. (2012). *National Mental Health Strategy 2012/13-2015/16*.

[13] Taylor R. S., Sander J. W., Taylor R. J., Baker G. A. (2011). Predictors of health-related quality of life and costs in adults with epilepsy: a systematic review. *Epilepsia*.

[14] Tegegne M. T., Muluneh N. Y., Wochamo T. T., Awoke A. A., Mossie T. B., Yesigat M. A. (2014). Assessment of quality of life and associated factors among people with epilepsy attending at Amanuel Mental Specialized Hospital, Addis Ababa Ethiopia. *Science Journal of Public Health*.

[15] George J., Kulkarni C., Sarma G. (2015). Antiepileptic drugs and quality of life in patients with epilepsy: a tertiary care hospital-based study. *Value in Health Regional Issues*.

[16] Anu M., Suresh K., Basavanna P. (2016). A cross-sectional study of quality of life among subjects with epilepsy attending a tertiary care hospital. *Journal of Clinical and Diagnostic Research*.

[17] Chen H.-F., Tsai Y.-F., Hsi M.-S., Chen J.-C. (2016). Factors affecting quality of life in adults with epilepsy in Taiwan: a cross-sectional, correlational study. *Epilepsy & Behavior*.

[18] Nagarathnam M., Vengamma B., Latheef S., Reddemma K. (2014). Assessment of quality of life in epilepsy in Andhra Pradesh. *NeurologyAsia*.

[19] Norsa’adah B., Zainab J., Knight A. (2013). The quality of life of people with epilepsy at a tertiary referral centre in Malaysia. *Health and Quality of Life Outcomes*.

[20] Jovel C. A. E., Salazar S. R., Rodríguez C. R., Mejía F. E. S. (2016). Factors associated with quality of life in a low-income population with epilepsy. *Epilepsy Research*.

[21] Sinha A., Sanyal D., Mallik S., Sengupta P., Dasgupta S. (2011). Factors associated with quality of life of patients with epilepsy attending a tertiary care hospital in Kolkata, India. *Neurology Asia*.

[22] Baker G. A., Jacoby A., Gorry J., Doughty J., Ellina V. (2005). Quality of life of people with epilepsy in Iran, the Gulf, and Near East. *Epilepsia*.

[23] Mosaku K. S., Fatoye F. O., Komolafe M., Lawal M., Ola B. A. (2006). Quality of life and associated factors among adults with epilepsy in Nigeria. *The International Journal of Psychiatry in Medicine*.

[24] Pimpalkhute S. A., Bajait C. S., Dakhale G. N., Sontakke S. D., Jaiswal K. M., Kinge P. (2015). Assessment of quality of life in epilepsy patients receiving anti-epileptic drugs in a tertiary care teaching hospital. *Indian Journal of Pharmacology*.

[25] Getnet A., Meseret S., Bekana L., Mekonen T., Fekadu W., Menberu M. (2016). Antiepileptic drug nonadherence and its predictors among people with epilepsy. *Behavioural Neurology*.

[26] Wakjira Rishe M. F. S., Belayneh K. G., Thirumurgan G., Esayas T., Gebremariam M. A. M.  (2015). Drug use evaluation of anti-epileptic drugs in out patient epilepsy clinic of Bishoftu general hospital, East Shoa, Ethiopia. *Indo American Journal of Pharmaceutical Research*.

[27] Birru E. M., Shafi M., Geta M. (2016). Drug therapy of epileptic seizures among adult epileptic outpatients of University of gondar referral and Teaching hospital, gondar, North West Ethiopia. *Dovepress*.

[28] Gurshaw M., Agalu A., Chanie T. (2014). Anti-epileptic drug utilization and treatment outcome among epileptic patients on follow-up in a resource poor setting. *Journal of Young Pharmacists*.

[29] Josephson C. B., Patten S. B., Bulloch A., Williams J. V., Lavorato D., Fiest K. M. (2017). The impact of seizures on epilepsy outcomes: a national, community-based survey. *Epilepsia*.

[30] Brusturean-Bota E., Coadă C. A., Buzoianu A. D., Perju-Dumbravă L. (2013). Assessment of quality of life in patients with epilepsy. *Human & Veterinary Medicine*.

[31] Singh P., Pandey A. K. (2017). Quality of life in epilepsy. *International Journal of Research in Medical Sciences*.

[32] Ashjazadeh N., Yadollahikhales G., Ayoobzadehshirazi A., Sadraii N., Hadi N. (2014). Comparison of the health-related quality of life between epileptic patients with partial and generalized seizure. *Iranian Journal of Neurology*.

[33] Wang F.-L., Gu X.-M., Hao B.-Y., Wang S., Chen Z.-J., Ding C.-Y. (2017). Influence of marital status on the quality of life of chinese adult patients with epilepsy. *Chinese Medical Journal*.

[34] Sajatovic M., Tatsuoka C., Welter E., Friedman D., Spruill T. M., Stoll S. (2017). Correlates of quality of life among individuals with epilepsy enrolled in self-management research: from the US Centers for Disease Control and Prevention Managing Epilepsy Well. *Network Epilepsy & Behavior*.

[35] Djibuti M., Shakarishvili R. (2003). Influence of clinical, demographic, and socioeconomic variables on quality of life in patients with epilepsy: findings from Georgian study. *Journal of Neurology, Neurosurgery & Psychiatry*.

[36] Thomas S. V., Koshy S., Nair C. S., Sarma S. P. (2005). Frequent seizures and polytherapy can impair quality of life in persons with epilepsy. *Neurology India*.

[37] Mutluay F. K., Gunduz A., Tekeoglu A., Oguz S., Yeni S. N. (2016). Health related quality of life in patients with epilepsy in Turkey. *Journal of Physical Therapy Science*.

